# Association of National Expansion of Insurance Coverage of Medically Tailored Meals With Estimated Hospitalizations and Health Care Expenditures in the US

**DOI:** 10.1001/jamanetworkopen.2022.36898

**Published:** 2022-10-17

**Authors:** Kurt Hager, Frederick P. Cudhea, John B. Wong, Seth A. Berkowitz, Sarah Downer, Brianna N. Lauren, Dariush Mozaffarian

**Affiliations:** 1 Friedman School of Nutrition Science and Policy, Tufts University, Boston, Massachusetts; 2 Division of Clinical Decision Making, Tufts Medical Center, Boston, Massachusetts; 3 Department of Medicine, Tufts University School of Medicine, Boston, Massachusetts; 4 Division of General Medicine and Clinical Epidemiology, Department of Medicine, University of North Carolina at Chapel Hill School of Medicine; 5 Cecil G. Sheps Center for Health Services Research, University of North Carolina at Chapel Hill; 6 Center for Medicare & Medicaid Innovation, Baltimore, Maryland; 7 Tufts University School of Medicine, Division of Cardiology at Tufts Medical Center, Boston, Massachusetts

## Abstract

**Importance:**

Medically tailored meals (MTMs) are associated with lower health care utilization among patients with complex diet-related diseases but are not a covered benefit in Medicare or Medicaid. The potential impact of extending insurance coverage for MTMs nationally remains unknown.

**Objective:**

To estimate 1- and 10-year potential changes in annual hospitalizations, potential changes in annual health care expenditures, and overall policy cost-effectiveness associated with national MTM coverage for US patients with diet-related disease and limited instrumental activities of daily living who have Medicaid, Medicare, or private insurance.

**Design, Setting, and Participants:**

In this economic evaluation, conducted from January 2021 to February 2022, a nationally representative sample from the 2019 Medical Expenditure Panel Survey was used to create a population-level cohort policy simulation model that estimated changes in annual hospitalizations and health care expenditures associated with coverage of MTMs. Participants were 6 309 998 US adults aged 18 years or older who had Medicare, Medicaid, or private payer insurance and at least 1 diet-sensitive condition and 1 limitation in instrumental activities of daily living.

**Interventions:**

Ten nutritionally tailored MTMs per week for a mean of 8 months in each year of intervention.

**Main Outcomes and Measures:**

The main outcomes were total hospitalizations, program costs, health care expenditures, and net policy costs. One thousand Monte Carlo simulations for each of 10 years (2019-2028) jointly incorporated uncertainty in model inputs for effect sizes, hospitalizations, health care expenditures, and program costs.

**Results:**

At the 2019 baseline, an estimated 6 309 998 US adults were eligible to receive MTMs. Mean (SD) age was 68.1 (16.6) years; most were female (63.4%), were non-Hispanic White (66.7%), and had Medicare and/or Medicaid (76.5%). The most common eligibility diagnoses were cardiovascular diseases (70.6%), diabetes (44.9%), and cancer (37.2%). If all eligible individuals received MTMs, an estimated 1 594 000 hospitalizations (95% uncertainty interval [UI], 1 297 000-1 912 000) and $38.7 billion (95% UI, $24.9 billion to $53.9 billion) in health care expenditures could potentially be averted in 1 year. Program costs were $24.8 billion (95% UI, $23.1 billion to $26.8 billion), for an associated net savings of $13.6 billion (95% UI, $0.2 billion to $28.5 billion) from a health care perspective. In 2019 dollars, 10 years of the MTM intervention was anticipated to cost $298.7 billion (95% UI, $279.7 billion to $317.4 billion) and to potentially be associated with 18 257 000 averted hospitalizations (95% UI, 14 690 000-22 109 000) and reductions in health care expenditures of $484.5 billion (95% UI, $310.2 billion to $678.4 billion), for net savings of $185.1 billion (95% UI, $12.9 billion to $377.8 billion). Findings were robust in multiple sensitivity analyses.

**Conclusions and Relevance:**

The findings suggest that national implementation of MTMs for patients with diet-sensitive conditions and activity limitations could potentially be associated with approximately 1.6 million averted hospitalizations and net cost savings of $13.6 billion annually. The results may inform US state, federal, and private-payer interest in expanding insurance coverage for MTMs among patients with diet-related chronic illness.

## Key Points

**Question:**

What potential changes in health care utilization and expenditures would be associated with national implementation of medically tailored meal (MTM) access for patients with diet-related diseases and limited instrumental activities of daily living who have Medicaid, Medicare, or private insurance?

**Findings:**

This economic evaluation among 6 309 998 eligible US adults found that national implementation of MTMs for patients with diet-sensitive conditions and activity limitations could potentially be associated with 1.6 million averted hospitalizations and net cost savings of $13.6 billion annually from an insurer perspective.

**Meaning:**

These findings suggest that national MTM coverage would be associated with cost savings for a range of circumstances if appropriately targeted.

## Teaser

This economic evaluation assesses the association of national expansion of medically tailored meals with health care utilization and expenditures among US patients with diet-related diseases and limited instrumental activities of daily living.

## Introduction

Despite a suboptimal diet being a leading factor associated with morbidity and mortality in the US,^1^ the health care system has traditionally had few tools to connect patients with chronic illness to nutrition services.^2^ The availability of nutrition programs is rapidly growing due to interest among health care systems, payers, patients, and policy makers in “food is medicine” interventions, such as medically tailored meals (MTMs), as potential tools to improve nutrition security, health outcomes, and health care utilization for these patients.^2,3^ Medically tailored meals are fully prepared, nutritionally tailored, and generally home-delivered healthy meals for individuals living with advanced and costly diet-sensitive conditions, such as diabetes, heart failure, end-stage kidney disease, HIV infection, and cancer. Programs generally provide 10 weekly meals (lunch and dinner for 5 days per week) designed by a registered dietitian based on disease diagnosis and nutritional assessment.^4^ Medically tailored meal programs are often designed to treat individuals with lower income, food insecurity, and/or limitations in instrumental activities of daily living (IADLs) that make it difficult to prepare healthy meals.

In observational studies and pilot randomized clinical trials, patients receiving MTMs experienced better disease management and had fewer hospitalizations, emergency department admissions, nursing home visits, and lower health care expenditures compared with similar control patients.^5,6,7,8,9,10^ Compared with no receipt of MTMs, MTM receipt has been associated with a 37% to 52% lower risk of hospitalization, 16% to 31% reduction in monthly health care expenditures,^5,6,7,8^ and decreased net costs of approximately $2500 per patient-year after paying for meal costs.^5,6^

Medically tailored meals, especially those evaluated in the literature, are generally provided by community-based organizations supported by grants, donations, and additional ad hoc restricted funding from home health care services benefits, Medicare Advantage programs, or state Section 1115 waivers allowing coverage of MTMs.^11,12^ Medically tailored meals are not currently a covered benefit in Medicaid or Medicare. Bills at both state and federal levels have recently proposed expanded access to MTMs in Medicaid and Medicare, but these have not passed.^13,14^ Availability of MTMs in federal programs depends on (1) whether regulatory flexibility or other special circumstances permit their inclusion and, if permitted, (2) whether participating private health care entities choose to cover them.^2,12^

Given the limited coverage for MTMs nationally, this treatment is unavailable to most US individuals who might benefit. To our knowledge, no previous research has modeled expected changes in health care expenditures and hospitalizations if MTMs were covered nationally by health insurance for the population routinely served by existing MTM organizations. The objective of this study was to estimate 1- and 10-year associations of MTMs with hospitalizations, health care expenditures, and net costs among patients with diet-related diseases and limitations in IADLs who are covered by Medicaid, Medicare, and private insurance.

## Methods

### Population and Setting

In this economic evaluation, the study sample was drawn from the 2019 Medical Expenditure Panel Survey (MEPS), a nationally representative survey of health care utilization and costs among noninstitutionalized US adults. The MEPS provides detailed individual-level data, including age, sex, race and ethnicity, family income, geographic region, IADL limitations, medical conditions, health care utilization, and costs. Race and ethnicity were assessed by self-report within the MEPS data set and were included to better understand the study sample and the potential implications expanded MTM coverage may have for health equity. This study was conducted from January 2021 to February 2022 and was approved by the Tufts Health Sciences Institutional Review Board, which deemed informed consent not applicable because the study was considered non–human participants research based on use of publicly available, deidentified data. We followed the Consolidated Health Economic Evaluation Reporting Standards (CHEERS) reporting guideline.

### Patient Eligibility

We modeled eligible patients as adults aged 18 years or older who were covered by Medicare, Medicaid, or private payers and had 1 or more diet-sensitive conditions and 1 or more IADL limitations. Diet-sensitive conditions included diabetes, congestive heart failure, myocardial infarction, other heart disease, emphysema, and stroke, as defined as priority conditions in the MEPS 2019 Combined File,^15^ and nonmelanoma cancer, chronic kidney disease, and HIV infection, as defined by *International Statistical Classification of Diseases and Related Health Problems, Tenth Revision* codes in the MEPS 2019 Medical Conditions File.^16,17^ These diagnoses were selected because they reflect the patient populations in previous MTM research^5,6,7,8,9,10,18,19,20^ and are served by the national network of MTM providers.^4^ The IADL limitations were defined as a positive survey response to receiving help or supervision using the telephone, paying bills, taking medications, preparing light meals, doing laundry, or going shopping due to an impairment or health problem.^15^

### MTM Intervention

We assumed meals were medically tailored and provided only for the index patient (ie, no meals were provided for other household members, as is common in most MTM insurance contracts). In practice, MTM organizations often create 10 to 15 meal plans per day, with registered dietitian nutritionists tailoring ratios of macronutrients and micronutrients for specific diagnoses, incorporating optimal quantities of healthy food groups such as fruits and vegetables, accounting for dietary preferences such as vegetarian options, and providing options for individuals who have challenges chewing solid foods. The MTM dietary guidelines for common diagnoses are provided on the Food Is Medicine Coalition website.^21^

### Statistical Analysis

#### MTM Duration

We conducted a PubMed search for studies that measured the association of MTMs with hospitalizations and/or health care expenditures in the US in the past 20 years and identified 5 studies.^5,6,7,8,22^ Using these studies, we calculated the weighted mean duration of MTM receipt as 8 months of MTMs per patient per year (eTable 1 in the Supplement). We also conducted an original meta-analysis to estimate policy effect sizes by pooling findings from published interventional studies^5,6,7,8,22^ using inverse variance-weighted meta-analysis with random effects. We conservatively assumed that benefits of MTMs would occur only in the year of MTM provision, with no sustained or carryover benefits into the following year.

#### Policy Costs

Annual MTM program costs included clinical screening costs and meal costs (Table 1). Screening costs were based on 2020 registered dietitian facility Medicare reimbursement rates for an initial, 15-minute medical nutritional therapy session at approximately $30 per patient (ranged from $27.34 in Mississippi to $35.17 in Santa Clara County, California).^23^ Meal costs were based on 2019 insurance contracts from 10 major MTM organizations and included nutritional tailoring, ingredients, and labor, administrative, and delivery costs. In our 1-year and 10-year models, we calculated net policy costs as the sum of changes in health care expenditures associated with MTM receipt and total MTM program costs.

**Table 1.** Inputs for Policy Simulation Model to Estimate 1-Year and 10-Year Change in Hospitalizations and Health Care Expenditures Associated With Provision of MTMs and Net Policy Costs

Input	Data source	Details
Annual inpatient admissions and health care expenditures stratified by insurance status	Medical Expenditure Panel Survey administered by the Agency for Healthcare Research and Quality^15^	Data source was used to identify model sample and estimate eligible population
Estimated policy effect sizes	Original meta-analysis of 5 published MTM studies that assessed associations of MTM receipt with inpatient hospitalizations and/or health care expenditures^5,6,7,8,22^^,^^a^	Effect sizes include pooled relative risks of inpatient hospitalizations and percentage change in health care expenditures
MTM costs from insurance contracts	Original survey to Food Is Medicine Coalition organizations^4^ administered from August to September 2021	Survey included questions about 2019 annual meals delivered, participants served, organization expenditures, and monthly meal costs from insurance contracts; 11 of the 15 organizations asked to complete the survey responded
Screening and referral costs	Medicare reimbursement rates for an initial medical nutrition therapy assessment by a registered dietitian nutritionist^23^	One-time added cost for each eligible MTM recipient in each year of the policy simulation model

Abbreviation: MTM, medically tailored meal.

^a^ Details are given in eTables 2 and 3 in the Supplement.

For the 10-year policy model, we assessed historical trends in population size and annual health care expenditures among MTM-eligible patients from 2010 to 2019 using MEPS data. We adjusted each year’s expenditures to 2019 US dollars^24^ using log-linear regression stratified by insurance status for the eligible population and used these inflation-adjusted trends to project the 2020 to 2028 expenditures and population size from the 2019 MEPS data. Given the specific patient population eligible for MTMs, we chose this empirical forecasting approach using MEPS data rather than incorporating expected trends for the general US population. For the 10-year model, we assumed all eligible participants received meals for 8 months per year in each year. We modeled an open cohort in which the population size for each year reflected people newly eligible entering and those who were no longer eligible leaving.

#### Simulation Model

We used a population-level, cohort policy simulation model to estimate the change in hospitalizations and health care expenditures that might occur after implementing an MTM policy compared with the status quo (ie, no new MTM policy), programmed in R, version 4.1.2 (R Project for Statistical Computing). For the 1-year (2019) and 10-year (2019-2028) time horizons, model outputs included changes in annual inpatient hospitalizations and health care expenditures, MTM program costs, and net policy costs from the health care perspective, separately analyzed among Medicaid, Medicare, dual-eligible, and privately insured patients. Model inputs included annual hospitalizations and expenditures from MEPS, relative risks of hospitalizations and the percentage change in health care expenditures associated with MTM receipt, and MTM program costs (Table 1). Medical Expenditure Panel Survey weights were used to scale the sample to be nationally representative.

Simulations were repeated 1000 times in each year so that probabilistic analyses jointly incorporated uncertainty in model inputs for hospitalizations, health care expenditures, effect sizes, and screening and meal costs by drawing randomly from within the plausible range of each input during simulation. Estimates reflect the mean of 1000 Monte Carlo simulations, with the 95% uncertainty interval (UI) defined as the 2.5th percentile to the 97.5th percentile of the simulations. Ten-year estimates summed separate simulations for each year (2019-2028) and assumed all eligible individuals received meals for 8 months in each year, with 3% annual discounting of health care expenditures and MTM program costs.

#### Secondary and Sensitivity Analyses

Because some MTM contracts condition receipt on experiencing food insecurity, a secondary analysis restricted the patient population by adding food insecurity as an eligibility criterion for the 1-year model (using 2017 MEPS data, the most recent data set including food insecurity). Food insecurity is defined by the US Department of Agriculture as “the limited or uncertain availability of nutritionally adequate and safe foods.”^25^ Multiple, 1-way sensitivity analyses assessed the robustness of findings to specific assumptions in the 1-year model, including estimating potential impacts for individuals with diabetes and congestive heart failure only, because these represent the most diet-sensitive conditions among our eligibility criteria. A third 1-year sensitivity analysis assumed only 50% of eligible patients received MTMs. A fourth sensitivity analysis estimated potential policy impact at the 2.5th 10th, 25th, 50th, 75th, 90th, and 97.5th percentiles of the effect size for change in health care expenditures associated with MTM receipt, holding constant all other inputs at their central estimate. Finally, we performed a threshold analysis to estimate the per-meal cost at which the overall 1-year net policy costs would break even (ie, be 0) and a separate threshold analysis to estimate the minimum change in health care expenditures associated with MTM receipt for the overall policy costs to break even, holding constant all other inputs at their central estimate. For the 10-year model, sensitivity analyses included alternative annual discounting rates of 0% and 5% and incorporating a second year of sustained reductions in health care expenditures among 15% of MTM recipients without requiring MTMs in the second year. All statistical analyses were done using R, version 4.1.2.

## Results

### Eligible Patients

We estimated that in 2019, 6 309 998 US adults with Medicare, Medicaid, and private insurance would have been eligible to receive MTMs based on having at least 1 diet-sensitive disease and IADL limitation (Table 2). Mean (SD) patient age was 68.1 (16.6) years; 63.4% were female, 36.6% were male, 11.3% were Hispanic, 3.1% were non-Hispanic Asian, 14.2% were non-Hispanic Black, 66.7% were non-Hispanic White, and 4.7% were other (specific groups are unavailable because this category was precoded within the MEPS data set) or multiple races and ethnicities. Median ratio of household income to poverty level was 1.9 (IQR, 0.9-3.7), and 76.5% of patients were covered by Medicare and/or Medicaid. The most common eligibility diagnosis was cardiovascular diseases (70.6%), followed by diabetes (44.9%) and cancer (37.2%) (Table 2). Mean (SD) annual health care expenditures in 2019 were $31 134 ($34 749) per person, including a mean (SD) of 0.98 (1.68) emergency department visits and 0.54 (0.94) hospitalizations per person per year, consistent with the expected high severity of illness and health care utilization in this patient population.

**Table 2.** Sample Description of Individuals Eligible to Receive Medically Tailored Meals in the US by Eligibility Criteria Using Data From the Medical Expenditure Panel Survey

Variable	Individuals^a^	
	Primary population (n = 6 309 998)^b^	Secondary population (n = 1 887 681)^c^
Age, mean (SD), y	68.1 (16.6)	60.5 (15.5)
Sex		
Female	4 001 494 (63.4)	1 202 453 (63.7)
Male	2 308 504 (36.6)	685 228 (36.3)
Race and ethnicity		
Hispanic	710 456 (11.3)	364 322 (19.3)
Non-Hispanic Asian	193 736 (3.1)	73 620 (3.9)
Non-Hispanic Black	898 846 (14.2)	479 471 (25.4)
Non-Hispanic White	4 208 527 (66.7)	851 344 (45.1)
Other or multiple^d^	298 434 (4.7)	118 924 (6.3)
Ratio of family income to poverty level		
Mean (SD)	2.8 (2.7)	2.6 (2.5)
Median (IQR)	1.9 (0.9-3.7)	1.3 (0.8-2.2)
US Census region		
Northeast	1 124 402 (17.8)	290 703 (15.4)
Midwest	1 273 829 (20.2)	575 742 (30.5)
South	2 487 704 (39.4)	683 341 (36.2)
West	1 424 064 (22.6)	337 895 (17.9)
Insurance status		
Private	1 485 365 (23.5)	352 996 (18.7)
Medicare	2 571 563 (40.7)	598 395 (31.7)
Medicaid	697 293 (11.1)	307 692 (16.3)
Dual eligible	1 555 779 (24.7)	628 598 (33.3)
Disease diagnosis^e^		
Diabetes	2 830 507 (44.9)	977 819 (51.8)
Congestive heart failure	1 693 284 (26.8)	366 210 (19.4)
Myocardial infarction	1 345 389 (21.3)	369 985 (19.6)
Other heart disease	2 304 133 (36.5)	734 308 (38.9)
Stroke	2 279 549 (36.1)	420 953 (22.3)
Cancer	2 345 823 (37.2)	458 706 (24.3)
Emphysema	672 761 (10.7)	303 917 (16.1)
Chronic kidney disease	82 030 (1.3)	30 203 (1.6)
HIV infection	37 860 (0.6)	22 652 (1.2)
Annual ED admissions, No.		
Mean (SD)	0.98 (1.68)	1.09 (1.85)
Median (IQR)	0 (0-1)	0 (0-2)
Annual hospitalizations, No.		
Mean (SD)	0.54 (0.94)	0.59 (1.02)
Median (IQR)	0 (0-1)	0 (0-1)
Annual health care expenditures, $		
Mean (SD)	31 134 (34 749)	33 634 (48 978)
Median (IQR)	20 107 (8856-38 965)	19 153 (6392-41 460)

Abbreviations: ED, emergency department; IADL, instrumental activities of daily living; MEPS, Medical Expenditure Panel Survey.

^a^ Data are presented as number (percentage) of individuals unless otherwise indicated.

^b^ Noninstitutionalized US adults with nutrition-sensitive disease and IADL limitations, based on 667 individuals in the 2019 MEPS survey.

^c^ Noninstitutionalized US adults with nutrition-sensitive disease, IADL limitations, and food insecurity, based on 244 individuals in the 2017 MEPS survey.

^d^ Race and ethnicity groups included as “other” are unavailable because the category was precoded within the MEPS data set.

^e^ Totals do not equal 100% because eligible individuals may have multiple comorbidities.

### Effect Sizes

In the meta-analysis of 5 previous studies,^5,6,7,8,22^ MTM provision was associated with reductions of annual health care expenditures of 19.7% (95% CI, 6.9%-32.4%) and annual hospitalizations of 47.0% (31.7%-62.3%) compared with usual care (eTables 2 and 3 in the Supplement). The pooled mean (SD) per-meal cost was $9.30 ($0.64).

### Estimated 1-Year Outcomes

If all eligible individuals received MTMs, the estimated MTM program costs, including clinical screening and meals, would be $24.8 billion (95% UI, $23.1 billion to $26.8 billion), and an estimated 1 594 000 hospitalizations (95% UI, 1 297 000-1 912 000) and $38.7 billion (95% UI, $24.9 billion to $53.9 billion) in health care expenditures would potentially be averted in 1 year (Table 3). Most of the health care expenditure savings (77.0%) would occur in Medicare and Medicaid, totaling $29.8 billion (95% UI, $22.2 billion to $38.2 billion). Summed across all health care payers, the policy was estimated to potentially be associated with net cost savings of $13.6 billion (95% UI, $0.2 billion to $28.5 billion). By payer subsets, 1-year possible policy cost savings were estimated at $3.1 billion (95% UI, −$2.9 billion to $9.5 billion) for private payers, $3.4 billion (95% UI, −$5.4 billion to $12.1 billion) for Medicare, $1.7 billion (95% UI, −$1.1 billion to $5.1 billion) for Medicaid, and $5.9 billion (95% UI, −$1.9 billion to $14.1 billion) for dual-eligible individuals. In probabilistic analyses combining all insurance strata, the intervention was associated with net cost savings in more than 97% of the simulations (Figure).

**Table 3.** Estimated 1-Year Averted Hospitalizations, Savings in Health Care Expenditures, and Net Policy Cost Savings Associated With Provision of MTMs, by Eligibility Criteria^a^

Insurance type	Individuals, No.	Averted annual inpatient hospitalizations, No. (95% UI)^b^	Savings in annual health care expenditures, $, in billions (95% UI)^c^	MTM program costs, $, in billions (95% UI)	Net policy cost savings, $, in billions (95% UI)
Noninstitutionalized US adults with nutrition-sensitive disease and IADL limitations					
Private	1 485 365	290 000 (173 000 to 419 000)	8.9 (2.7 to 15.7)	5.9 (5.1 to 6.7)	3.0 (−2.8 to 9.4)
Medicare	2 571 562	712 000 (455 000 to 1 013 000)	13.4 (4.4 to 22.7)	10.1 (8.8 to 11.6)	3.4 (−5.4 to 12.1)
Medicaid	697 292	195 000 (102 000 to 327 000)	4.5 (1.5 to 8.0)	2.8 (2.4 to 3.2)	1.7 (−1.1 to 5.1)
Dual eligible	1 555 779	397 000 (241 000 to 579 000)	11.9 (4.0 to 20.7)	6.1 (5.3 to 7.0)	5.9 (−1.9 to 14.1)
Total	6 309 998	1 594 000 (1 297 000 to 1 912 000)	38.7 (24.9 to 53.9)	24.8 (23.1 to 26.8)	13.6 (0.2 to 28.5)
**Noninstitutionalized US adults with nutrition-sensitive disease, IADL limitations, and food insecurity**					
Private	330 587	78 000 (37 000 to 126 000)	2.7 (0.5 to 5.8)	1.3 (1.1 to 1.5)	1.4 (0.7 to 4.3)
Medicare	587 828	167 000 (86 000 to 272 000)	3.1 (1.0 to 5.5)	2.3 (2.0 to 2.6)	0.8 (−1.3 to 3.2)
Medicaid	286 066	117 000 (76 000 to 171 000)	2.8 (0.6 to 5.8)	1.1 (0.9 to 1.3)	1.7 (−0.4 to 4.6)
Dual eligible	683 200	144 000 (76 000 to 228 000)	4.4 (1.4 to 8.0)	2.7 (2.3 to 3.0)	1.8 (−1.1 to 5.3)
Total	1 887 681	506 000 (398 000 to 654 000)	13.0 (7.9 to 18.9)	7.4 (6.9 to 8.0)	5.5 (0.7 to 11.1)
**Noninstitutionalized US adults with diabetes and IADL limitations**					
Private	636 320	118 000 (62 000 to 183 000)	4.3 (1.2 to 7.9)	1.9 (2.1 to 1.6)	2.4 (−0.7 to 6.1)
Medicare	1 001 345	304 000 (180 000 to 463 000)	5.6 (1.9 to 9.5)	3.0 (2.5 to 3.4)	2.6 (−1.2 to 6.5)
Medicaid	368 460	63 000 (24 000 to 120 000)	2.5 (0.7 to 4.6)	1.1 (0.9 to 1.2)	1.4 (−0.3 to 3.5)
Dual eligible	824 381	216 000 (123 000 to 326 000)	7.0 (2.3 to 12.4)	2.4 (2.1 to 2.8)	4.6 (−0.1 to 10.0)
Total	2 830 506	701 000 (524 000 to 911 000)	19.3 (12.2 to 27.3)	8.4 (7.8 to 9.1)	10.9 (3.6 to 18.8)
**Noninstitutionalized US adults with congestive heart failure and IADL limitations**					
Private	374 445	77 000 (31 000 to 128 000)	2.5 (0.7 to 4.5)	1.1 (0.9 to 1.3)	1.4 (−0.4 to 3.4)
Medicare	871 058	288 000 (168 000 to 436 000)	5.0 (1.7 to 8.7)	2.6 (2.2 to 2.9)	2.4 (−0.8 to 6.1)
Medicaid	119 035	37 900 (24 000 to 57 000)	0.7 (0.2 to 1.5)	0.4 (0.3 to 0.4)	0.4 (−0.1 to 1.1)
Dual eligible	330 745	127 000 (68 000 to 196 000)	2.6 (0.8 to 5.0)	1.0 (0.8 to 1.1)	1.6 (−0.2 to 4.0)
Total	1 695 294	530 000 (373 000 to 705 000)	10.9 (6.3 to 15.6)	5.0 (4.6 to 5.4)	5.8 (1.3 to 10.6)

Abbreviations: IADL, instrumental activities of daily living; MTM, medically tailored meal; UI, uncertainty interval.

^a^ Estimates are the mean of 1000 Monte Carlo simulations, with the 95% UI defined as the 2.5th percentile to the 97.5th percentile of the simulations. The policy simulation model ran 1000 Monte Carlo simulations using inputs and their uncertainties from the 2019 Medical Expenditure Panel Survey, relative risks of annual hospitalizations, and annual percentage change in health care expenditures associated with MTM receipt, screening costs, and meal costs.

^b^ Rounded to the nearest 1000.

^c^ Rounded to the nearest $100 000 000.

**Figure.**
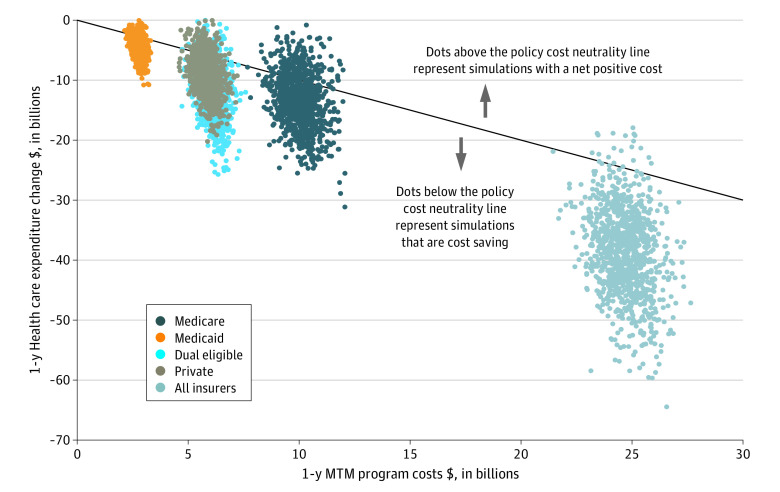
Model Simulations of 1-Year Medically Tailored Meal (MTM) Policy Costs and Potential Change in Health Care Expenditures Associated with MTM Receipt, by Health Insurance Status Each dot represents 1 of 1000 simulations, stratified by insurance status. The diagonal line indicates policy cost neutrality. The policy simulation model ran 1000 Monte Carlo simulations using inputs and their uncertainties from the 2019 Medical Expenditure Panel Survey, relative risks of annual hospitalizations, and annual percentage change in health care expenditures associated with MTM receipt, screening costs, and meal costs.

### One-Year Sensitivity Analyses

When adding food insecurity as an eligibility criterion, eligibility decreased from 6 309 998 to 1 887 681 individuals. Compared with the original population, the smaller population was younger and more likely to be Hispanic or non-Hispanic Black, have lower household income, and be dual-eligible for Medicare and Medicaid (Table 2). Baseline annual emergency department admissions, hospitalizations, and health care expenditures were slightly greater than for the base-case population, although these differences were not statistically significant. In the smaller population, our model estimated that implementation of MTMs would potentially be associated with 506 000 averted hospitalizations (95% UI, 398 000-654 000) and $13.0 billion (95% UI, $7.9 billion to $18.9 billion) averted health care expenditures in 1 year, with a net policy cost savings of $5.5 billion (95% UI, $0.7 billion to $11.1 billion) (Table 3).

A total of 2 830 507 individuals in the primary population (44.9%) had diabetes. In this population, our model estimated that MTMs could potentially be associated with 701 000 averted hospitalizations (95% UI, 524 000-911 000) and $19.3 billion (95% UI, $12.2 billion to $27.3 billion) averted health care expenditures in 1 year, with a net policy cost savings of $10.9 billion (95% UI, $3.6 billion to $18.8 billion). A total of 1 695 294 individuals had congestive heart failure (26.8%), and in this population, our model estimated that MTM receipt was associated with a potential reduction of 530 000 hospitalizations (95% UI, 373 000-705 000) and $10.9 billion (95% UI, $6.3 billion to $15.6 billion) health care expenditures in 1 year, with a net policy cost savings of $5.8 billion (95% UI, $1.3 billion to $10.6 billion). The 3 secondary populations were anticipated to have between 139% and 173% greater per capita net policy cost savings than the primary population (eTable 4 in the Supplement).

Assuming only 50% coverage of eligible participants in the primary population, the policy was estimated to potentially be associated with 798 000 averted hospitalizations (95% UI, 648 500-956 000) and $19.4 billion (95% UI, $12.5 billion to $26.9 billion) averted health care expenditures, with a net policy cost savings of $6.90 billion (95% UI, $0.10 billion to $14.20 billion) in 1 year. For the 1-year policy to be cost neutral rather than cost saving, the estimated per-meal cost would need to double from $9.30 to $18.89, and the change in health care expenditures associated with MTM receipt would need to decrease by more than one-third, from 19.7% to 12.7%. Across the uncertainty range from the 2.5th to 97.5th percentile of the effect size for change in health care expenditures associated with MTM receipt, most scenarios would be anticipated to be cost saving, with the potential for significantly greater benefits than are reported in our primary findings (eFigure in the Supplement). However, it remains possible that the policy may have a net positive cost if the true effect of MTM receipt on change in health care expenditures is below the 14th percentile of our effect size uncertainty range.

### Estimated 10-Year Outcomes

Based on observed national trends with 2019 as the base, we assumed that from 2020 to 2028, the eligible patient population would increase annually by 1.0% among privately insured individuals, 2.1% among those in Medicare, 3.0% among those in Medicaid, and 5.7% among dual-eligible individuals and that per-patient, inflation-adjusted health care expenditures would increase by 1.5% annually among privately insured individuals, 1.7% among those in Medicare, 3.5% among those in Medicaid, and 3.9% among dual-eligible individuals. In 2019 dollars, 10 years of the MTM intervention, in which the target population received MTMs for 8 months per year in each of the 10 years modeled, was estimated to potentially cost $298.7 billion (95% UI, $279.7 billion to $317.4 billion) and to be associated with reductions in hospitalizations of 18 257 000 (95% UI, 14 690 000-22 109 000) (Table 4) and in health care expenditures of $484.5 billion (95% UI, $310.2 billion to $678.4 billion) (eTable 5 in the Supplement). The net cost savings from an insurer perspective would be $185.1 billion (95% UI, $12.9 billion to $377.8 billion) if the target population received MTMs for 8 months per year in each of the 10 years modeled (Table 4).

**Table 4.** Estimated 10-Year Averted Hospitalizations and Net Policy Cost Savings Associated With Provision of MTMs, by Discounting Approach^a^

Insurance type	10-y Averted hospitalizations, No. (95% UI)^b^	10-y Net policy cost savings, 2019 $, in billions (95% UI)^c^		
		No discounting of future costs	3% Discounting of future costs^d^	5% Discounting of future costs
Private	3 029 000 (1 812 000 to 4 385 000)	62.2 (−27.1 to 156.4)	45.5 (−32.8 to 127.3)	36.0 (−34.0 to 110.4)
Medicare	7 836 000 (5 008 000 to 11 144 000)	52.1 (−62.4 to 164.7)	30.2 (−69.4 to 128.2)	17.6 (−73.3 to 107.4)
Medicaid	2 226 000 (1 169 000 to 3 748 000)	31.1 (−12.1 to 81.5)	22.6 (−14.8 to 66.2)	17.7 (−16.4 to 57.4)
Dual eligible	5 166 000 (3 132 000 to 7 519 000)	115.2 (−16.9 to 260.0)	88.0 (−27.0 to 213.5)	72.4 (−32.0 to 186.6)
Total	18 257 000 (14 690 000 to 22 109 000)	260.7 (62.7 to 481.5)	185.1 (12.9 to 377.8)	143.7 (−11.8 to 319.4)

Abbreviations: MTM, medically tailored meal; UI, uncertainty interval.

^a^ In each of the 10 years, the eligible population was assumed to receive 8 months of medically tailored meals per year. Estimates are the mean of 1000 Monte Carlo simulations, with the 95% UI defined as the 2.5th percentile to the 97.5th percentile of the simulations. The policy simulation model ran 1000 Monte Carlo simulations using inputs and their uncertainties from the 2019 Medical Expenditure Panel Survey, relative risks of annual hospitalizations, and annual percentage change in health care expenditures, MTM receipt, screening costs, and meal costs. The policy simulation model was run separately and then summed for each of the 10 years (2019-2028) to obtain final estimates. Baseline distributions of hospitalizations and health care expenditures from 2020 to 2028 were estimated using the historical rate of change in population size and health care expenditures from 2010 to 2019 for the target population.

^b^ Rounded to the nearest 1000.

^c^ Rounded to the nearest $100 000 000.

^d^ Primary analysis.

### Ten-Year Sensitivity Analyses

Applying either 5.0% discounting or no discounting of costs, $441.2 billion (95% UI, $282.7 billion to $617.7 billion) or $558.4 billion (95% UI, $357.3 billion to $782.1 billion), respectively, in health care expenditures would potentially be adverted (eTable 5 in the Supplement). The net 10-year cost savings were $143.7 billion (95 % UI, −$11.8 billion to $319.4 billion) for 5.0% discounting and $260.7 billion (95% UI, $62.7 billion to $481.5 billion) for no discounting (Table 4). Assuming benefits of MTMs persisted into a second year for 15% of individuals (and with 3% discounting), the estimated 10-year potential net cost savings were $231.5 billion (95% UI, $41.2 billion to $441.4 billion).

## Discussion

Combining nationally representative data on patient eligibility and health care utilization with evidence from interventional studies of MTMs, our simulation model estimated that full national coverage of MTMs in Medicare, Medicaid, and private insurance for patients with both a diet-sensitive condition and an IADL limitation would be associated with meaningfully reductions in annual hospitalizations and health care expenditures. Among 6 309 998 eligible recipients, MTMs could possibly be associated with a reduction of 1 594 000 hospitalizations annually. Furthermore, after accounting for the costs of identifying and referring patients to MTM organizations and providing 10 weekly meals for a mean of 8 months annually, the policy was anticipated to be associated with a net savings of $13.6 billion over 1 year and $185.1 billion over 10 years. These findings were robust to a range of sensitivity and scenario analyses.

### Alignment With Prior Research

The provision of prepared meals through health care first arose as a palliative measure for patients with AIDS under the Ryan White Comprehensive AIDS Resources Emergency Act of 1990, at a time when few effective treatments for HIV infection existed. The potential utility of MTMs in clinical care is now supported by interventional studies observing improved diet quality, food security, and disease management when patients with diet-sensitive conditions receive MTMs.^8,9,10,20,26^ Medically tailored meals have been associated with reduced depressive symptoms and fewer dilemmas among paying for food, health care, or prescriptions.^20^ Receipt of MTMs is also associated with improved disease management. For example, among patients with HIV infection receiving MTMs, antiretroviral therapy adherence increased, and among patients with diabetes, diabetes self-management improved.^20,26^ These prior findings suggest that MTMs may be associated with improved health through several pathways, including improved nutrition, improved food security, better financial well-being, reduced stress and anxiety, and improved medication adherence and self-management.

Other research supports additional mechanisms by which MTMs are associated with reduced hospitalizations and lower health care expenditures. For example, patients with advanced cirrhosis and ascites required fewer weekly paracenteses and experienced improved ascites-specific quality of life after 3 months of MTM receipt.^9^ Among patients with recent hospitalization for heart failure, 1 month of MTM receipt was associated with improved clinical symptom and quality-of-life scores on the Kansas City Cardiomyopathy Questionnaire.^8^ In a recent randomized clinical trial, 600 patients hospitalized with chronic heart failure were assigned to receive either usual hospital meals or medically tailored meal plans, nutritional counseling, and if necessary, supplemental intravenous nutrition. The tailored nutritional support led to a 56% reduction in mortality at 30 days.^27^ Although meals were provided in the hospital rather than delivered at home, this trial supports the benefits of comprehensive, tailored nutritional support for patients with chronic illness who are at high risk of hospitalization.

### Interpretation of Results

We modeled MTM provision to patients with severe comorbidities and limitations to independence, and these findings should not be generalized to a healthier population or to a less intensive nutrition intervention. We do not anticipate similar results for individuals with the same diagnoses who do not have limited IADLs. Our main analysis was based on full coverage of all eligible individuals to provide a best-case policy scenario. In practice, it would take time for MTM services to scale and serve all eligible patients. Thus, our scenario analysis of 50% coverage is an alternative benchmark for comparison. Scaling of MTMs may be associated with increased program efficiency and reduced costs, leading to greater net savings. Conversely, scaling may be associated with lower nutritional quality or tailoring of meals, reducing efficacy. These possibilities need to be evaluated with empirical research. Nonetheless, the robustness of our results suggests that national MTM coverage may be cost saving under a range of circumstances if appropriately targeted, including adding food insecurity as a criterion for eligibility or focusing only on patients with diabetes or congestive heart failure. Sensitivity analyses suggest that further targeting to these higher-need populations may be associated with greater per capita cost savings but lower population-level cost savings because fewer people are eligible. Finally, the primary goal of MTMs is to provide high-quality medical care for patients with chronic illness, and the observed reductions in hospitalizations and expenditures do not incorporate potential additional benefits in patient-related quality of life, disease progression, caregiver well-being, and population-level health equity.

### Health Policy Relevance

This investigation leveraged national data and interventional findings to estimate the health and economic impacts of MTM expansion within Medicaid, Medicare, and private insurance. The findings support policy expansion of access to MTMs by adopting health policy reforms. Several states are currently piloting expanded MTM access, including a $6 million pilot in California for patients with heart failure^28^; a $149 million pilot of flexible services in Massachusetts that covers nutrition and housing programs including MTMs among patients with Medicaid^29^; and a similar $650 million Section 1115 waiver in North Carolina’s Medicaid program that allows payment for MTMs.^30^ Multiple private payers are experimenting with MTMs through charitable donations and grants.^4^ Kaiser Permanente, the largest health maintenance organization in the US, is undertaking a large clinical trial of MTMs for chronically ill patients.^31^ At the federal level, the Medically Tailored Home-Delivered Meals Demonstration Pilot Act of 2021 has been introduced to direct Medicare to implement and evaluate MTMs, and similar bills have recently been introduced at the state level.^13,14^ As of 2020, Medicare Advantage plans may also choose to provide MTMs to certain beneficiaries. Despite this accelerating use of MTMs, access depends on buy-in from state-level Medicaid administrators or managed care plan leadership and is therefore limited by geographic region and/or insurance carriers. The current limitations in national coverage of MTMs present an opportunity to improve health if future policies expand MTM access, with an additional opportunity to improve health equity if such policies prioritize patients with low income and/or food insecurity. Our findings support the use of MTM programs and the need for their timely implementation, scaling, and evaluation in both public and private health care.

The COVID-19 pandemic has further highlighted the need to invest in prevention and treatment of nutrition-sensitive chronic conditions, with a focus on health equity. The US Centers for Disease Control and Prevention has documented diet-related conditions including diabetes, cardiovascular diseases, chronic kidney disease, cancers, and obesity as leading risk factors for COVID-19 hospitalizations and deaths.^32^ Our research suggests that expanding MTM access should be considered as a health care strategy to improve care for patients with diet-related health conditions.

### Strengths and Limitations

This study has strengths. We incorporated national data on eligible patients and health care utilization and expenditures, increasing generalizability of the findings. Effect sizes were derived from interventional studies of MTMs and MTM program costs from insurance contracts between MTM providers and health care systems. Patient eligibility criteria were consistent with prior research studies and existing MTM programs. Our policy model included probabilistic sensitivity analyses across 1000 Monte Carlo simulations to jointly incorporate uncertainty and report a range of plausible outcomes, and additional 1-way sensitivity analyses tested the effects of specific assumptions on the results. We estimated both 1-year and 10-year outcomes, providing a range of clinically relevant and policy-relevant time horizons.

The study also has limitations. Although all included MTM studies were interventional, most were observational, with carefully constructed rather than randomized groups. However, existing randomized clinical trials of MTMs that assessed other health outcomes^8,9,10,27^ support the estimated benefits of interventions shown in observational studies. The published literature was insufficient to incorporate other outcomes that may be impacted by MTMs, such as emergency department admissions, nursing home admissions, and patient quality of life. Published MTM evaluations^5,6^ report up to 2 years of intervention, and our 10-year estimates could be either overestimates or underestimates of cost savings, depending on whether MTM efficacy strengthens or wanes over time. The eligible population analyzed represents an open cohort, with some newly entering eligible patients and others exiting due to mortality or loss of eligibility. In addition, our base-case 10-year analysis assumed no carryover benefits of receiving MTMs; thus, policy benefits may have been underestimated if some MTM recipients had lasting improvements in health after 1 year of intervention.

## Conclusions

Our simulation model of a nationally representative MTM-eligible population with diet-sensitive conditions and IADL limitations estimated that coverage for MTMs in Medicare, Medicaid, and private insurance could potentially be associated with approximately 1.6 million averted hospitalizations and net savings of $13.6 billion in health care costs in the first year. These findings may inform increasing state, federal, and private-payer interest in implementing “food is medicine” interventions such as MTMs to address diet-related chronic illness in the US.

## Notes

Supplement. **eFigure.** Net Annual Policy Costs by Percentile of Effect Size for Reductions in Health Care Expenditures Associated With Medically Tailored Meal Receipt **eTable 1.** Weighted Average of the Observation Period and MTM Intervention Lengths in Previous Studies **eTable 2.** Meta-analysis of Previous Studies That Assessed the Impact of MTM Receipt on Inpatient Hospitalizations **eTable 3.** Meta-analysis of Previous Studies That Assessed the Impact of MTM Receipt on Health Care Expenditures **eTable 4.** Per Capita, 1-Year Estimated Averted Hospitalizations, Savings in Health Care Expenditures, and Net Policy Cost Savings Attributable to Provision of MTMs, by Eligible Population **eTable 5.** Ten-Year Savings in Health Care Expenditures Attributable to MTM Receipt, by Discounting Approach
eReferences
