# Supplementary material for: Association of National Expansion of Insurance Coverage of Medically Tailored Meals With Estimated Hospitalizations and Health Care Expenditures in the US
Source: JAMA Netw Open. 2022 Oct 17;5(10):e2236898. doi: 10.1001/jamanetworkopen.2022.36898 (PMC9577678; doi:10.1001/jamanetworkopen.2022.36898)
Supplement: Supplement. — eFigure. Net Annual Policy Costs by Percentile of Effect Size for Reductions in Health Care Expenditures Associated With Medically Tailored Meal Receipt eTable 1. Weighted Average of the Observation Period and MTM Intervention Lengths in Previous Studies eTable 2. Meta-analysis of Previous Studies That Assessed the Impact of MTM Receipt on Inpatient Hospitalizations eTable 3. Meta-analysis of Previous Studies That Assessed the Impact of MTM Receipt on Health Care Expenditures eTable 4. Per Capita, 1-Year Estimated Averted Hospitalizations, Savings in Health Care Expenditures, and Net Policy Cost Savings Attributable to Provision of MTMs, by Eligible Population eTable 5. Ten-Year Savings in Health Care Expenditures Attributable to MTM Receipt, by Discounting Approach eReferences [file jamanetwopen-e2236898-s001.pdf]

Supplementary Online Content

Hager K, Cudhea FP, Wong JB, et al. Association of national expansion of insurance coverage of medically tailored meals with estimated hospitalizations and health care expenditures in the US. *JAMA Netw Open*. 2022;5(10):e2236898. doi:10.1001/jamanetworkopen.2022.36898

eFigure. Net Annual Policy Costs by Percentile of Effect Size for Reductions in Health Care Expenditures Associated With Medically Tailored Meal Receipt

eTable 1. Weighted Average of the Observation Period and MTM Intervention Lengths in Previous Studies

eTable 2. Meta-analysis of Previous Studies That Assessed the Impact of MTM Receipt on Inpatient Hospitalizations

eTable 3. Meta-analysis of Previous Studies That Assessed the Impact of MTM Receipt on Health Care Expenditures

eTable 4. Per Capita, 1-Year Estimated Averted Hospitalizations, Savings in Health Care Expenditures, and Net Policy Cost Savings Attributable to Provision of MTMs, by Eligible Population

eTable 5. Ten-Year Savings in Health Care Expenditures Attributable to MTM Receipt, by Discounting Approach

eReferences

This supplementary material has been provided by the authors to give readers additional information about their work.

© 2022 Hager K et al. *JAMA Network Open*.

eFigure. Net Annual Policy Costs by Percentile of Effect Size for Reductions in Health Care Expenditures Associated With Medically Tailored Meal Receipt

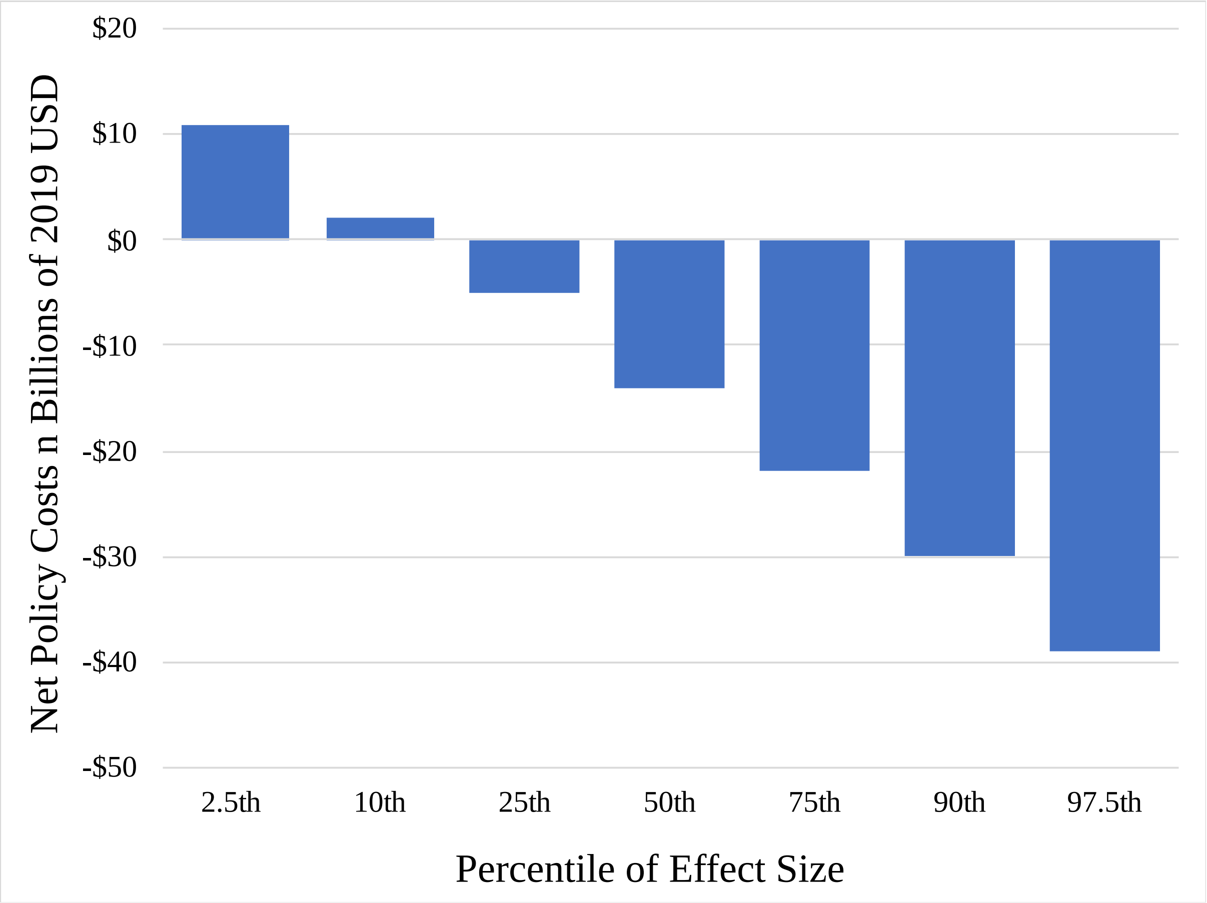

Percentiles are equivalent to the following one-year change in healthcare expenditures associated with eight months MTM receipt: 2.5th = -6.9%; 10th = -11.4%; 25th = -15.3%; 50th= -19.7% (central estimate); 75th= -24.1%; 90th= -28.0%; 97,5th = -32.4%. Healthcare expenditures would need to be reduced by 12.6% for the policy to be cost neutral, equivalent to the 14th percentile of the effect size uncertainty range for change in healthcare costs associated with MTM receipt. Effect size estimates are from an original metanalysis of all known studies assessing the association between MTM receipt and inpatient hospitalizations and/or healthcare expenditures conducted in the U.S. in the past 20 years.

© 2022 Hager K et al. *JAMA Network Open*.

eTable 1. Weighted Average of the Observation Period and MTM Intervention Lengths in Previous Studies

Study	Number of MTM recipients in the intervention group	Observation Period	MTM Intervention Length
**Berkowitz 1** [1]	499	21.4 months	12.4 months
**Berkowitz 2** [2]	133	19.1 months	17.8 months
**Gurvey** [3]	65	6.0 months	6.0 months
**Hummel** [4]	33	3.0 months	1.0 month
**Weighted Average**	**730**	**18.8 months**	**12.2 months**

The weighted average observation time and MTM intervention length included all known studies assessing the association between MTM receipt and inpatient hospitalizations and/or healthcare expenditures conducted in the U.S. in the past 20 years. The study by Horton[5] did not specify the average observation period nor intervention length.

© 2022 Hager K et al. *JAMA Network Open*.

eTable 2. Meta-analysis of Previous Studies That Assessed the Impact of MTM Receipt on Inpatient Hospitalizations

Study	Relative Risk of Inpatient Hospitalization Associated with MTM receipt	Standard Error
Berkowitz 1 [1]	0.51	0.16
Berkowitz 2 [2]	0.48	0.16
Hummel [4]	0.68	0.19
Gurvey [3]	0.51	0.13
**Pooled Effect**	**0.53**	**0.08**

Results from inverse variance meta-analysis with random effects that included all known studies assessing the association between MTM receipt and inpatient hospitalizations and/or healthcare expenditure in the U.S. in the past 20 years. Only one study[2] reported the impact of MTMs on emergency department admissions (in a second study,[3] the authors stated that their results for emergency department admissions were "inconclusive"). Therefore, we did not incorporate MTM impacts on emergency department admissions in our analysis as they would have been dependent on a single study.

© 2022 Hager K et al. *JAMA Network Open*.

eTable 3. Meta-analysis of Previous Studies That Assessed the Impact of MTM Receipt on Health Care Expenditures

**Study**	**Percent Change in Annual Healthcare Expenditures Associated with MTM receipt**	**Standard Error**
Berkowitz 1 [1]	-17.0%	5.3%
Berkowitz 2 [2]	-16.0%	5.7%
Horton [5]	-24.0%	8.3%
Gurvey [3]	-31.0%	9.0%
**Pooled Effect**	**-19.7%**	**6.5%**

Results from inverse variance meta-analysis with random effects that included all known studies assessing the association between MTM receipt and inpatient hospitalizations and/or healthcare expenditure in the U.S. in the past 20 years.

© 2022 Hager K et al. *JAMA Network Open*.

eTable 4. Per Capita, 1-Year Estimated Averted Hospitalizations, Savings in Health Care Expenditures, and Net Policy Cost Savings Attributable to Provision of MTMs, by Eligible Population

Insurance	Population Size	Per Capita Averted Inpatient Hospitalizations (95% UI)	Per Capita Savings in Healthcare Expenditures (95% UI)	Per Capita Net Policy Cost Savings
Primary Population: Non-institutionalized US adults with nutrition sensitive disease, and IADL limitations				
Private	1,485,365	0.19 (0.12, 0.28)	$5,920 (1,800, 10,560)	$2,090 (-1,960, 6,390)
Medicare	2,571,562	0.28 (0.18, 0.40)	$5,250 (1,710, 8,820)	$1,320 (-2,110, 4,710)
Medicaid	697,292	0.28 (0.15, 0.47)	$6,310 (2,150, 11,470)	$2,490 (-1,600, 7,270)
Dual eligible	1,555,779	0.26 (0.15, 0.37)	$7,460 (2,570, 13,300)	$3,770 (-1,200, 9,100)
**Total**	**6,309,998**	**0.25 (0.15, 0.37)**	**$6,090 (3,940, 8,540)**	**$2,230 (50, 4,530)**
Non-institutionalized US adults with nutrition sensitive disease, IADL limitations, and food insecurity				
Private	330,587	0.23 (0.11, 0.38)	$7,860 (1,490, 17,380)	$4,168 (-1,280, 10,170)
Medicare	587,828	0.28 (0.15, 0.46)	$5,100 (1,700, 9,350)	$4,160 (-1,380, 10,400)
Medicaid	286,066	0.41 (0.26, 0.60)	$9,440 (2,090, 20,270)	$1,290 (-640, 3,860)
Dual eligible	683,200	0.21 (0.11, 0.33)	$6,290 (2,050, 11,790)	$2,410 (-230, 5,790)
**Total**	**1,887,681**	**0.27 (0.14, 0.42)**	**$6,670 (4,180, 10,010)**	**$3,090 (690, 5,602)**

© 2022 Hager K et al. *JAMA Network Open*.

Non-institutionalized US adults with diabetes and IADL limitations				
Private	**636,320**	0.19 (0.10, 0.29)	$6,740 (1,880, 12,370)	$3,770 (-1,050, 9,580)
Medicare	**1,001,345**	0.30 (0.18, 0.46)	$5,560 (1,870, 9,480)	$2,580 (-1,200, 6,450)
Medicaid	**368,460**	0.17 (0.07, 0.33)	$6,660 ($1,960, $12,550)	$3,700 (-910, 9,560)
Dual eligible	**824,381**	0.26 (0.15, 0.40)	$8,550 (2,770, 14,990)	$5,580 (-160, 12,140)
**Total**	**2,830,506**	**0.25 (0.19, 0.32)**	**$6,838 (4,330, 9,650)**	**$3,870 (1,290, 6,660)**
Non-institutionalized US adults with congestive heart failure and IADL limitations				
Private	374,445	0.21 (0.08, 0.34)	$6,650 (1,904, 12,120)	$3,680 (-1,130, 8,970)
Medicare	871,058	0.33 (0.19, 0.50)	$5,780 (1,970, 10,010)	$2,800 (-931, 7,010)
Medicaid	119,035	0.31 (0.20, 0.48)	$6,080 (1,450, 12,300)	$3,110 (-1,534, 9,280)
Dual eligible	330,745	0.38 (0.21, 0.59)	$7,950 (2,400, 15,040)	$4,970 (-470, 11,970)
**Total**	**1,695,293**	**0.31 (0.22, 0.42)**	**$6,420 (3,770, 9,210)**	**$3,443 (-770, 6,238)**

Estimates are the mean of 1,000 Monte Carlo simulations with the 95% uncertainty interval defined as the 2.5th percentile to the 97.5th percentile of the simulations. The policy simulation model runs 1,000 Monte Carlo simulations using inputs and their uncertainties from 2019 Medical Expenditure Panel Survey, relative risks of annual hospitalizations and annual percent change in healthcare expenditures associated MTM receipt, screening costs and meal costs.

© 2022 Hager K et al. *JAMA Network Open*.

eTable 5. Ten-Year Savings in Health Care Expenditures Attributable to MTM Receipt, by Discounting Approach

**Insurance**	*No Discounting of Future Costs*<br><br>**10-Year Savings in Healthcare Expenditures in Billions of 2019 USD** (95% UI)	*3% Discounting of Future Costs (primary analysis)*<br><br>**10-Year Savings in Healthcare Expenditures in Billions of 2019 USD** (95% UI)	*5% Discounting of Future Costs*<br><br>**10-Year Savings in Healthcare Expenditures in Billions of 2019 USD** (95% UI)
Private	$125.9 (38.2, 220.8)	$109.2 (33.3, 191.7)	$99.6 (30.2, 174.7)
Medicare	$168.7 (56.0, 285.6)	$146.9 (48.7, 248.6)	$134.3 (44.5, 227.0)
Medicaid	$64.2 (20.9, 114.8)	$55.8 (18.2, 99.7)	$50.7 (16.6, 90.8)
Dual eligible	$199.6 (67.2, 346.8)	$172.6 (58.1, 299.9)	$156.8 (52.8, 272.5)
**Total**	**$558.4 (357.3, 782.1)**	**$484.5 (310.2, 678.4)**	**$441.2 (282.7, 617.7)**

In each of the ten years, the eligible population was assumed to receive 8 months of medically tailored meals per year. This table reports potential savings in healthcare expenditures only and does report the net policy costs. Estimates are the mean of 1,000 Monte Carlo simulations with the 95% uncertainty interval defined as the 2.5th percentile to the 97.5th percentile of the simulations. The policy simulation model runs 1,000 Monte Carlo simulations using inputs and their uncertainties from 2019 Medical Expenditure Panel Survey, relative risks of annual hospitalizations and annual percent change in healthcare expenditures MTM receipt, screening costs and meal costs. The policy simulation model was run separately and then summed for each of the ten years (2019-2028) to obtain final estimates. Baseline distributions of hospitalizations and healthcare expenditures for years 2020-2028 were estimated using the historical rate of change in population size and healthcare expenditures from 2010-2019 for the target population. Healthcare expenditures are rounded to the nearest $100,000,000.

© 2022 Hager K et al. *JAMA Network Open*.

eReferences

1. Berkowitz SA, Terranova J, Randall L, Cranston K, Waters DB, Hsu J. Association between receipt of a medically tailored meal program and health care use. *JAMA Intern Med*. 2019;179(6):786-793. Medline:31009050 doi:10.1001/jamainternmed.2019.0198

2. Berkowitz SA, Terranova J, Hill C, et al. Meal delivery programs reduce the use of costly health care in dually eligible Medicare and Medicaid beneficiaries. *Health Aff (Millwood)*. 2018;37(4):535-542. Medline:29608345 doi:10.1377/hlthaff.2017.0999

3. Gurvey J, Rand K, Daugherty S, Dinger C, Schmeling J, Laverty N. Examining health care costs among MANNA clients and a comparison group. *J Prim Care Community Health*. 2013;4(4):311-317. Medline:23799677 doi:10.1177/2150131913490737

4. Hummel SL, Karmally W, Gillespie BW, et al. Home-delivered meals postdischarge from heart failure hospitalization. *Circ Heart Fail*. 2018;11(8):e004886. Medline:30354562 doi:10.1161/CIRCHEARTFAILURE.117.004886

5. Horton R, Paulson K, Aaron D, Raab R, Hardy RY, Pulling E. Medically tailored meals reduce medical costs for chronically ill. 2018. Accessed September 15, 2022. https://www.projectangelheart.org/food-is-medicine/research-policy/small-intervention-big-impact/

© 2022 Hager K et al. *JAMA Network Open*.
